# Root puppet masters: Infauna shift trait‐productivity relationships in submerged aquatic vegetation communities

**DOI:** 10.1002/ece3.70305

**Published:** 2024-10-25

**Authors:** Charlotte Angove, Alf Norkko, Camilla Gustafsson

**Affiliations:** ^1^ Stable Isotope Laboratory of Luke (SILL) Natural Resources Institute Finland (Luke) Helsinki Finland; ^2^ Tvärminne Zoological Station University of Helsinki Hanko Finland

**Keywords:** facilitation, functional traits, infauna, SAV, seagrass

## Abstract

Submerged aquatic vegetation (SAV) growth can be limited by light and nutrient availability. Infauna are common inhabitants of SAV meadows. Their activity increases nutrient mobility, and they can positively affect plant growth, but we do not know their role in plant trait‐biomass production relationships. We approached this problem using a 15‐week in situ transplant experiment in the Baltic Sea with experimental additions of *Macoma balthica*, a sedentary bivalve, to experimental SAV communities. Experimental plant communities were tricultures with varying species composition, compiled from a pool of six different species, to create an experimental gradient of trait community weighted means that allowed us to detect changes more clearly in plant trait‐biomass production relationships in response to the *M. balthica* treatment. We evaluated the relationships between plant height, leaf area, maximum root length (MMRL), specific root length (SRL), and SAV biomass production, then compared *M. balthica* condition index (soft tissue biomass [WW, mg]/valve length [mm]) to plant community leaf tissue nutrient concentrations (N (%DW), δ^15^N). Community biomass production was significantly related to plant height in the control treatment, but this relationship was decoupled in the *M. balthica* treatment, where community biomass production was instead significantly related to MMRL and SRL. This suggested a shift in the predominant SAV growth strategy, from height‐related to root‐related community biomass production. Leaf tissue δ^15^N was significantly related to *M. balthica* condition index. The growth of one species, *Potamogeton perfoliatus*, was significantly inhibited by the *M. balthica* treatment. Our results show that infauna have an important role in the plant traits related to community biomass production, and they have the potential to shape plant community structure via selective influences on different plant species.

## Graphical abstract

While co‐restoration of seagrass and infauna is a viable solution to increase submerged aquatic vegetation (SAV) restoration success, more research is needed to understand how infauna affects the drivers of SAV biomass production. In this study, we conducted a transplant experiment with synthetic mixed‐species SAV communities and an infauna treatment, to explore how infauna can affect plant trait‐productivity relationships. Results showed that infauna can change plant trait‐productivity relationships by decoupling of the plant height‐biomass production relationship and stronger relationships between plant biomass production and nutrient acquisition traits.

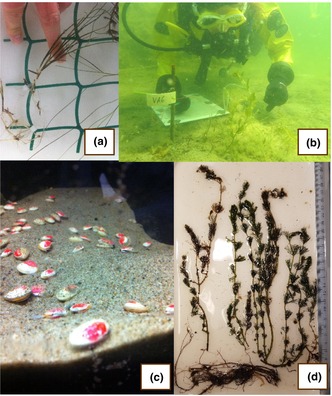

## 1 INTRODUCTION

Two of the most limiting factors to affect submerged aquatic vegetation (SAV) growth are light attenuation and nutrient availability (Lee et al., 2007). Light attenuation can affect the depth limit of SAV (Ralph et al., 2007) and, generally, SAV are found more in areas with higher light penetration and lower nutrient concentrations compared to more nutrient‐rich ecosystems which have a lower light penetration (Krause‐Jensen et al., 2008). Benthic infauna are common components of SAV meadows (Boström et al., 2006), therefore it is essential to understand the facilitative relationship between infauna and SAV to holistically understand SAV ecology (Duffy, 2006). Infauna can have major roles in sediment nutrient cycling (Norkko et al., 2013), and macrofaunal symbionts can enrich SAV with nutrients (Peterson & Heck, 1999, 2001), which otherwise might not be provided by the nutrient‐poor environment (Angove et al., 2018; Carroll et al., 2008), therefore they can have a considerable role for SAV biomass production (e.g., Cardini et al., 2022; Peterson & Heck, 2001). Infauna are being explored as potential natural solutions to aid seagrass restoration and seagrass resilience to future climate change (Cardini et al., 2022; Gagnon et al., 2020), and they could be increasingly important as the climate warms (Clemente & Thomsen, 2023). Therefore, it is essential to better understand why infauna additions are not always beneficial to seagrass biomass production (e.g., Meysick et al. (2020)) by, for example, understanding how infauna affect the relationships between traits manifested by SAV and their biomass production.

A wide variety of macroinvertebrate infauna inhabit temperate SAV meadows; for example, sessile bivalves, burrowing oligochaetes, polychaetes, and crustaceans (Creed & Sheldon, 1995; Fredriksen et al., 2010; Gammal et al., 2019). Infauna can have positive effects on subtidal seagrass, with potential benefits to plant survivability, growth, and resilience (Gagnon et al., 2020). The known mechanisms by which infauna benefit SAV are nutrient enrichment, increased water clarity and lowered sulphide stress (Carroll et al., 2008; Gagnon et al., 2020; Reynolds et al., 2007; van der Geest et al., 2020; van der Heide et al., 2012; Wall et al., 2008). There could be further, undiscovered benefits to submerged aquatic plant ecosystem multifunctionality, which have been quantified for salt marshes (Angelini et al., 2015), based on the combined outcome of decomposition, soil accretion, invertebrate biomass, infiltration rate, aboveground biomass, and benthic algae biomass.

A plant functional trait is a heritable morpho‐physio‐phenological characteristic which can enhance a process related to plant fitness, such as biomass production (Garnier et al., 2016; Kattge et al., 2011; Violle et al., 2007). Plant functional traits are well‐established tools for investigating mechanistic interactions between plants and their environment (Levine, 2016; Pérez‐Harguindeguy et al., 2013). In submerged temperate aquatic communities, the traits plant height and leaf area are strongly related to productivity, likely because they enhance light capture, as well as increase plant biomass per unit area and enhance competitive community interactions for light capture (Angove et al., 2020; Gustafsson & Norkko, 2019). However, SAV growth can be limited by carbon (Buapet et al., 2013), or nitrogen (e.g., van Lent et al., 1995) availability in temperate marine environments with siliclastic sediments, that is, sediments consisting of silica or silicates. Evidence from the northern Baltic Sea suggests that there is a plant nutrient demand driven by plant biomass, based on a negative correlation between plant biomass and sediment porewater ammonium (${\mathrm{NH}}_{4}^{+}$) concentrations (Angove et al., 2018). As a result, SAV with traits that help absorb nutrient enrichment by infauna might proliferate more in the presence of infauna, meaning that infauna would have a role in determining the plant traits which are most beneficial for SAV growth.

One of the most prevalent macroinvertebrate infauna species in northern Baltic Sea sediments is the Baltic tellin, *Macoma balthica*, which is a sedentary bivalve found in soft sediment environments (Bonsdorff et al., 1995) that plays a major role in the recycling of nutrients through their metabolic activities, feeding behaviour and influence on sediment redox conditions (Norkko et al., 2013). They are typically found 0–4 cm deep in the sediment, and they randomly diffuse particles around them; because they move particles in a random manner and over short distances (Bernard et al., 2019; Michaud et al., 2005). They enrich the sediment with organic carbon by defecation and inorganic carbon by respiration (Ebenhöh et al., 1995), using organic matter and nutrients which they collect from the sediment–water interface (de Goeij & Luttikhuizen, 1998). As hosts of archaeal methanogenic symbionts, they also release significant quantities of methane (Bonaglia et al., 2017). Additionally, *M. balthica* oxygenate the sediment by increasing water flow from the oxygenated water column into the sediment (e.g., Michaud et al., 2005), and they release numerous other nutrients into the sediment and water column by excretion (e.g., Braeckman et al., 2010; Norkko et al., 2013). Therefore, it is highly likely that *M. balthica* affects plant trait‐productivity relationships; for example, by increasing nutrient supply and changing the traits most advantageous for optimising photosynthesis via the balance between investment in light versus nutrient acquisition.

We hypothesised that experimental additions of *M. balthica* to SAV communities would enhance plant biomass production (H1) by providing them with nutrients in the sediment which are not otherwise accessible to the plant roots (Angove et al., 2018; Carroll et al., 2008). We predicted that communities with *M. balthica* additions would exhibit a positive relationship between root fineness (i.e., specific root length, hereafter SRL) and plant community biomass production (H2), because plants with finer roots can reduce the diffusive distance between roots and the *M. balthica* nutrient efflux more effectively (Pérez‐Harguindeguy et al., 2013). Meanwhile, plant height and leaf area would be closely related to community production in both treatments (H3) because they are important in the region (Angove et al., 2020; Gustafsson & Norkko, 2019), and the clam addition could enhance their importance since nutrient enrichment can increase aboveground biomass (Lee & Dunton, 2000; van Lent et al., 1995) and plant leaf area (Lee & Dunton, 2000; Peterson & Heck, 2001). We expected to find a reciprocal positive effect to *M. balthica* by plant communities, due to the potential increased food supply and shelter from predators (Gagnon et al., 2020). Subsequently, since *M. balthica* condition indices are indicators of bivalve physiological status (Brylawski, 2009; Lucas & Beninger, 1985; Pierścieniak et al., 2010), we hypothesised that plant leaf tissue δ^15^N would decrease with increasing condition index of *M. balthica* (H4) (Handley & Raven, 1992), to indicate that the plants sourced nitrogen from the activity of the transplanted clams.

## 2 METHODS

### 2.1 Study area and experimental setup

Kyan (59.827, 23.21 WGS 84) is a shallow (depth of experiment = 2.5 m) brackish‐water lagoon in the Gulf of Finland, and it is inhabited by a diverse meadow of mixed plant species, amongst bare patches of sand. At the peak of the growing season in 2018, shoot density of vegetated patches at Kyan ranged from 125 to 2675 shoots m^−2^ (unpublished data). In the local region, SAV does not necessarily host higher densities of *M. balthica* in the sediment compared to bare sand patches (Meysick et al., 2019). Amongst the bare sand and vegetated patches in the region, *M. balthica* densities can range from 722 to 2708 individuals per square metre ($\bar{x}$ = 1119 ind. m^−2^) (Gammal et al., 2019). The adult (>5 mm) fraction of the population comprises 20% of the abundance (i.e. 200 ind. m^−2^) and 99% of the biomass. Alongside *M. balthica*, other dominant species of infauna are polychaetes; *Marenzelleria* spp. ($\bar{x}$ = 1697 ind. m^−2^), *Pygospio elegans* ($\bar{x}$ = 1444 ind. m^−2^) and *Hediste diversicolor* ($\bar{x}$ = 505 ind. m^−2^) (Gammal et al., 2019).

An in‐situ transplant experiment was conducted at Kyan using SCUBA in 2016, for the purpose of exploring the relationships between plant functional traits, plant functional diversity, and biomass production (Angove et al., 2020). This previously published data served as a control treatment for our simultaneous experiment, while both studies pursued different research questions with unique data analyses. We used a mark‐recapture approach with *M. balthica* in artificial plant communities identical to those in Angove et al. (2020), to observe if the clam additions changed plant trait‐productivity relationships. We used the same experimental blocks (6 × 8 × 4 m grids), and the setup is presented in Figure S1. The six experimental blocks were prepared on naturally occurring bare sand areas amongst the meadow. Infrequent plant shoots in the sandy areas indicated that the sediment was hospitable to plants.

The experimental plant community assemblage was a triculture of three different plant species, and species were randomly combined to achieve an experimental treatment of different heritable plant trait means which varied along a continuous scale. By constructing an experimental treatment of plant traits which varied along a continuous scale, it was possible to investigate relationships between plant traits and community biomass production using multivariate regression analysis. Importantly, the communities assembled were identical for the control treatment and the *M. balthica* treatment, which allowed for direct comparison in biomass production and trait values without plant species artefacts to results (Figure S1). The species pool for experimental communities comprised *Zostera marina*, *Potamogeton perfoliatus*, *Zannichellia major*, *Ruppia cirrhosa*, *Stuckenia pectinata* and *Myriophyllum spicatum*. Overall, there were 12 unique triculture combinations, each represented in at least two of the six different blocks. A full list is available in Table S1. In total, there were 42 experimental communities used for this study. Of these, there were 18 artificial plant communities used as control treatments and 18 artificial plant communities used for this experimental treatment. One final plot in each experimental block was a bare‐sand treatment with added clams (⅀ = 6), where plant‐assembly grids were buried without plants attached (Figure S1). These were used for comparing *M. balthica* biomass production between plant‐occupied and bare sand plots.

### 2.2 Experimental design

**FIGURE 1:**
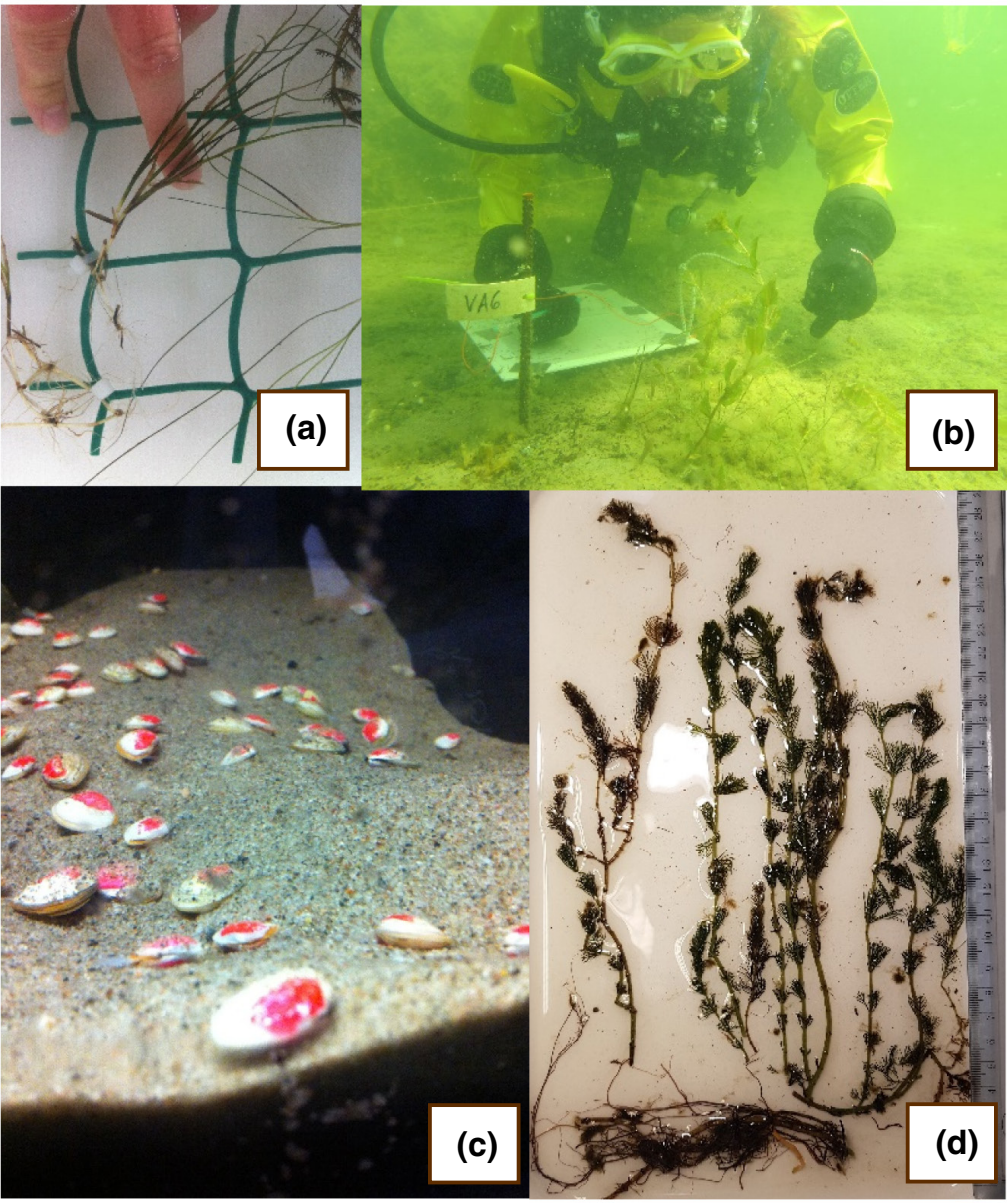
(a) Aquatic plant shoot attached to 30 × 30 cm grid to make artificial plant community (image credit Charlotte Angove). (b) Experimental community monitoring (image credit Tiina Salo). (c) Marked clams reburying themselves in an aquarium, during the reburial test before their transplant to experimental communities (image credit Charlotte Angove). (d) Example of grown shoots harvested from experimental communities (*Myriophyllum*, image credit Charlotte Angove).

At the start of the growing season, plants were collected intact, by stirring the sand and carefully extracting them from the sediment. We constructed experimental plant communities of 24 shoots on plastic grids (30 × 30 cm) using cable ties, while keeping the plants submerged (Figure 1). Within these experimental communities, there were eight shoots of each species, and the location of each shoot on the plot was assigned using a random number generator. During community assembly, we subsampled ten individuals of each species for an estimated start biomass. We transplanted the communities by massaging them into the sand carefully, then secured them using two stainless steel hooks. The experiment commenced on 01 June 2016, and after 2 weeks, we surveyed plant survival rate. While most of the time, transplants were successful, if an individual of a species was lost, we corrected the start biomass of the species. There were no species losses, therefore all plots were successfully tricultures throughout the experiment.

We used a mark‐recapture approach with *M. balthi*ca. Prior to transplantation, we collected ca. 300 individuals from the experimental site. We selected adult individuals (5–20 mm valve length), and then marked their valves using non‐toxic cosmetic red nail polish. After marking, the individuals who reburied themselves in aquarium conditions overnight were used for the transplant (Figure 1). A subsample of 30 *M. balthica* individuals was kept for trait measurements prior to the experiment. On 21 June 2016, we transplanted 10 *M. balthica* individuals to the centre of each treatment plot by partly inserting them into the sediment with their anterior side facing downwards to aid burial. Given that the plots were relatively small (30 × 30 cm), it was manageable to plant ten individuals in the centre to increase the likelihood of their recapture. The transplanted *M. balthica* were additions to naturally occurring background *M. balthica* density to avoid severe disturbance to the seafloor by removal of natural abundances (e.g., Meysick et al., 2020), therefore our treatment corresponded to an additional 111 ind. m^−2^ to natural total abundances ($\bar{x}$ = 1119 ind. m^−2^) and 30% increase in adult fraction of the population (Gammal et al., 2019). A higher density would have increased the risk of plant growth inhibition, as observed in the Finnish archipelago by Meysick et al. (2020). Our transplanted individuals inhabited sediment close to the root zone. Every 2–3 weeks, we revisited plots to monitor shoot production and remove weeds. After the 12‐week experiment, communities were harvested, on 14 September 2016. Water turbidity during the harvest would not allow us to recapture every clam for trait measurements and it is plausible that some individuals had either moved out of the plot or died. Nevertheless, on average, we retrieved 5–6 of the 10 *M. balthica* individuals that we originally transplanted.

### 2.3 Productivity and trait measurements

Immediately after sampling of plant communities, we subsampled new leaf tissue nutrient samples from the plants (Angove et al., 2020) and froze the remaining plant samples at −18°C for future processing. We did not measure porewater nutrient concentrations because standalone porewater concentrations are not representative of the entire sediment nutrient production (Mcglathery et al., 2001). We suspended the recaptured *M. balthica* in an aquarium with filtered seawater overnight using nylon bags before freezing them (−18°C) for future processing.

For each species in a community, we measured a variety of plant traits which were potentially important for primary production (Table 1; Figure 1), more details on measurement protocol are described in Angove et al. (2020). After trait measurements, we separated, oven‐dried (60°C, at least 48 h) and weighed all biomass pools for each species in each community: Leaves, aboveground rhizomes, belowground rhizomes and roots. We also measured *M. balthica* valve length (mm), soft tissue biomass (wet weight [WW], mg), then we used these measurements to calculate condition index (soft tissue biomass [WW, mg]/valve length [mm]).

**TABLE 1:** Focus submerged aquatic plant traits and their descriptions.

Plant trait	Description
Height (cm)	Length of intact shoot aboveground tissue, from 10 individuals until cumulative average analyses confirmed lower, species‐specific frequency requirements for height estimates
Leaf area (mm^2^)	Area of largest intact leaves from 5 individuals, excluding leaf sheaths
MMRL (cm)	Median Maximum Root Length, represented by median primary root length, excluding lengths of branches. Length of 10 longest primary roots from 5 individuals (⅀ ≥ 30)
SRL	Specific Root Length, an index of root fineness. The sum of root lengths used to find MMRL, divided by their collective dried weight (mm mg^−1^). Includes branch lengths
Leaf tissue δ^15^N (‰)	^15^N isotope ratio compared to ^14^N (VSMOW, ‰), in new leaf tissue
Leaf tissue N (% Dry Weight)	New leaf tissue percent nitrogen content

### 2.4 Data processing

We prepared and analysed data using R (R Core Team, 2022). Biomass production (mg Dry Weight [DW] day^−1^) was the increase in biomass (mg DW) throughout the experimental period (105 days), first calculated for each plant species in each community. We calculated the start biomass for each species by multiplying the number of successfully transplanted individuals by mean start biomass per individual, using weights from individuals subsampled at the start of the experiment. Community biomass production was the sum of species biomass production for each community. Height, maximum root length, and leaf area were summarised as species medians rather than species means, so that the presence of one or two exceptionally large specimens, did not bias the estimated average for the entire species in a plot. Community Weighted Means (CWMs) were based on the relative shoot frequencies of each species in a community (Garnier et al., 2004; Gustafsson & Norkko, 2019). Owing to the fragility of the studied plant leaves and roots, sometimes a representative measurement for a trait was lost, and handling of missing data is described for statistical analyses.

### 2.5 Statistical analyses

We used *t*‐tests to explore potential *M. balthica* treatment effects on trait CWMs, species biomass production and overall community biomass production, between paired experimental plots. For species comparisons, we used all complete paired species measurements, and for the community comparison, we used all paired plots with complete biomass measurements for all species. With non‐paired, Mann–Whitney *U* tests we compared *M. balthica* condition indices between the plant community treatment, bare sand treatment and prior to transplantation. For gradient tests which compared productivity to trait CWMs along a continuous scale, we used all measured trait data available, which resulted in an unequal group size for each treatment but the best representation of trait‐productivity relationships for that treatment from the largest possible sample size.

We used Spearman's correlation coefficient with a False Discovery Rate (Benjamini & Hochberg, 1995) to confirm that there were not strong (*r* > .7) significant relationships between traits which could have introduced Type I errors. We evaluated the combined relationship of height, SRL and MMRL community‐weighted means to community biomass production, using multiple linear regressions, for each of the control treatment and the *M. balthica* treatment. We tested for autocorrelation effects by experimental block identity, using generalised linear mixed models with block identity as random intercepts, which accounted for our experiment blocking design. However, since the quantified effect of block identity to results was so low (Intraclass correlation <.07), we used simple multiple regressions for parsimony. Since leaf area was strongly correlated to height (Spearman's rank: *r* = .77, *p* < .05), we excluded it from the multivariate analysis so that height represented light capture investment, and the relationship between leaf area and community biomass production was evaluated separately using linear regressions. Finally, we used linear mixed models with Block ID as a random intercept, to compare plot‐averaged *M. balthica* condition index (mean frequency per plot = 6) and with leaf tissue δ^15^N (‰) and N (% DW) community weighted means (CWMs). By comparing *M. balthica* condition index to plant leaf tissue nutrient concentrations along a continuous scale (gradient design), it was possible to explore the maximum potential effects of *M. balthica* on plant leaf nutrition.

## 3 RESULTS

### 3.1 Plant biomass response to *M. balthica* additions

**FIGURE 2:**
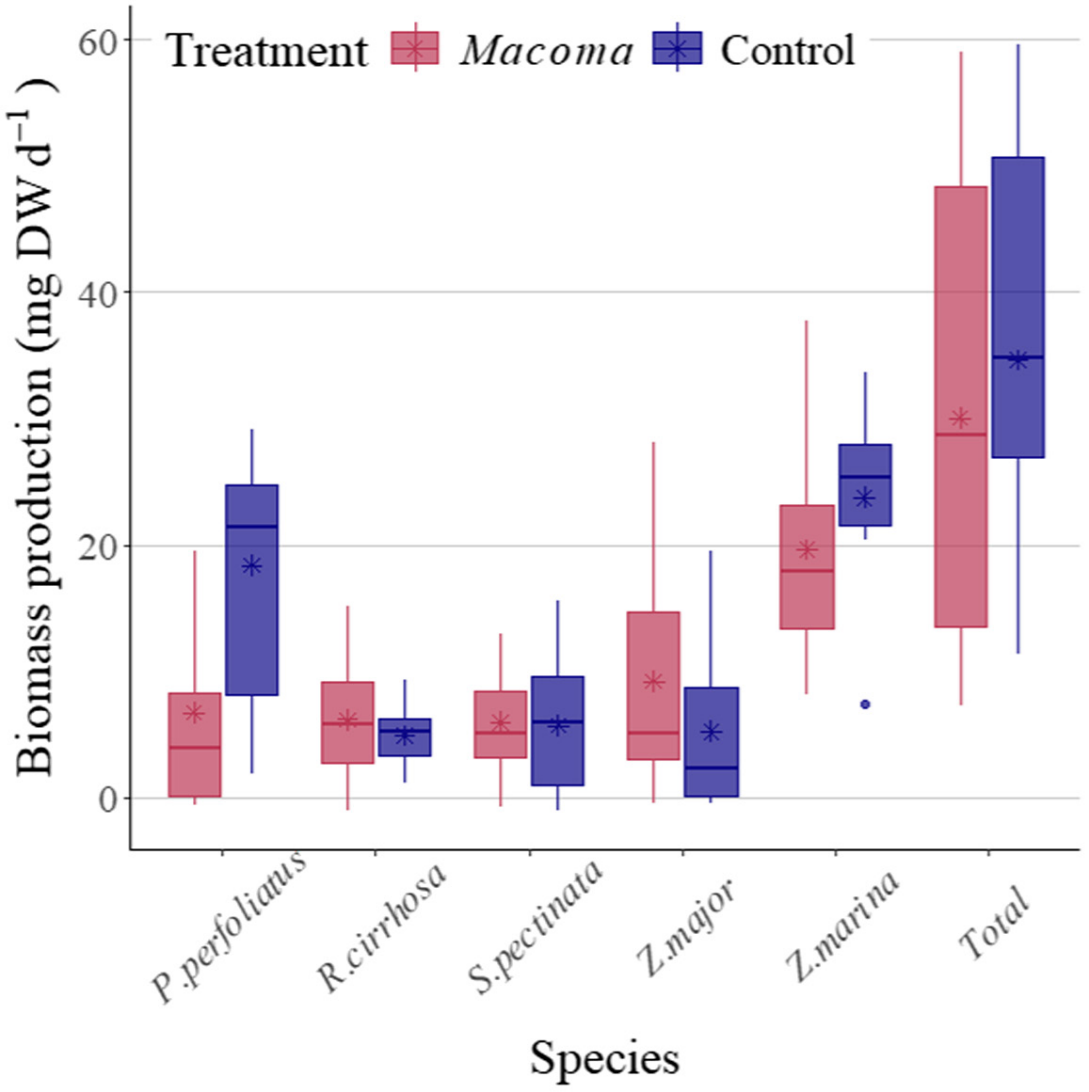
Species biomass production (mg dry weight day^−1^), and total community biomass production (‘Total’, mg DW day^−1^), from experimental plant communities with added *Macoma balthica* (‘*Macoma*’) and paired communities without added *M. balthica* (‘Control’), in the Finnish archipelago of the northern Baltic Sea. Coloured asterisks show means, and the black asterisk identifies a significant difference (*p* < .01).

The mean plant biomass production of communities with *M. balthica* additions (30.1 mg dry weight day^−1^) was not significantly different to the biomass production of paired control plant communities (34.6 mg DW day^−1^, *t*[25] = −0.682, *p* > .05). However, species responded differently to the *M. balthica* treatment: *P. perfoliatus* produced significantly less biomass in the *M. balthica* treatment than the control data (Figure 2, *t*[16] = −2.79, *p* < .01). Meanwhile, the other species did not significantly respond in biomass to the treatment: *R. cirrhosa* (Figure 2, *t*[14] = −0.61, *p* > .05), *S. pectinata* (*t*[16] = 0.117, *p* > .05), and *Z. marina* (*t*[15] = −0.451, *p* > .05). The biomass production of *Z. major* was not normally distributed in the control treatment (Shapiro–Wilk, *p <* .05), but there was not a significant rank difference between treatments (*W* = 35, *p* > .05). *M. spicatum* was too rare in the experiment for species‐specific, statistical comparison (*n* = 4).

### 3.2 Community trait‐productivity relationship response to *M. balthica* additions

**FIGURE 3:**
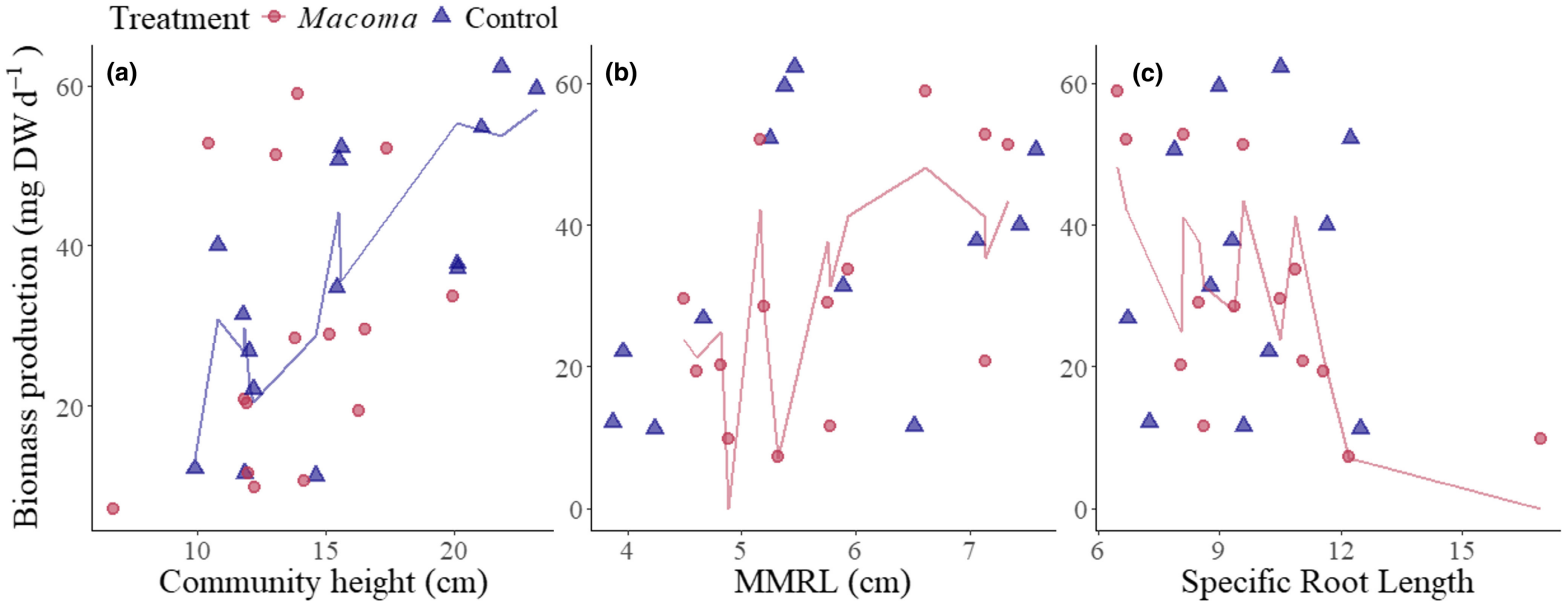
Plant community biomass production (mg DW day^−1^), in relation to the following community‐weighted mean traits: (a) Height (cm), (b) Median Maximum Root Length (MMRL, cm), (c) Specific Root Length (SRL), for mixed species aquatic plant plots with no added *Macoma balthica* (Control series) and with added *M. balthica* (*Macoma* series) in the northern Baltic Sea. Lines show multiple linear regression fits when a significant relationship is present, and they vary because of the influence of other traits in the multiple regression model.

**FIGURE 4:**
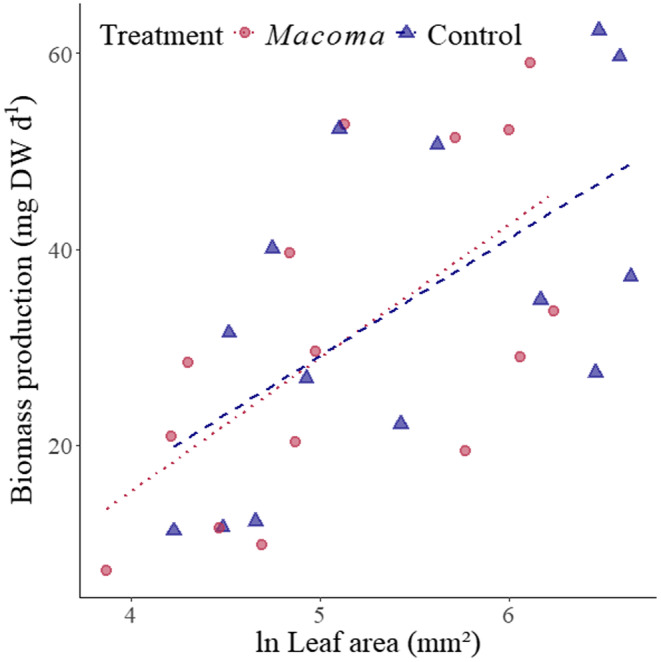
Relationship between ln‐transformed, community‐weighted mean leaf area (mm^2^), and plant community biomass production (mg DW day^−1^), for mixed species aquatic plant plots with no added *Macoma balthica* (Control series) and with added *M. balthica* (*Macoma* series) in the northern Baltic Sea.

The *M. balthica* treatment significantly affected plant trait‐productivity relationships (Figure 3; Table 3), without changing plant trait CWMs (Table S2). Multiple regression analyses both moderately described plant community biomass production in the *M. balthica* treatment and the control treatment (*R* ^2^ = .56 and *R* ^2^ = .47, respectively, Table 3). There was an evident change in the relationships between plant community traits and plant community biomass production (Figure 3; Table 2). For instance, in control plots, community height was the only trait significantly related to community biomass production, of the three traits selected for multivariate analyses (Figure 3a; Table 2). Meanwhile, the biomass production of communities with *M. balthica* additions was instead significantly related to community Median Maximum Root Length (MMRL, cm) and Specific Root Length (SRL) (Figure 3b,c; Table 2). This meant, that communities with longer, thicker, and denser roots were significantly more productive in response to the *M. balthica* treatment but not in the control treatment. Community leaf area was significantly related to community biomass production in the *M. balthica* treatment (Figure 4; Table 3).

**TABLE 2:** Multiple regression fit for the relationships between plant trait community weighted means (CWMs) and plant community biomass production (mg DW day^−1^), for communities with *Macoma balthica* transplantations (*n* = 13) and control communities (*n* = 8), when measurements for all traits are available.

Treatment	Trait	Coefficient	Intercept	Adj. *R* ^2^	*F*	*p*
*M. balthica* treatment	Height	1.92	−14.6	.56	6.62	**.01**
	MMRL	**8.33**				
	SRL	**−2.92**				
Control	Height	**2.82**	−31.21	.47	4.23	**.046**
	MMRL	4.07				
	SRL	0.12				

*Note*: Bold coefficient values show an individual trait is significant (*p* < .05, *t*‐statistic).

Abbreviations: MMRL, median maximum root length; SRL, specific root length.

**TABLE 3:** Linear regression statistics for the relationship between ln‐transformed, community‐weighted mean leaf area (mm^2^), and plant community biomass production (mg DW day^−1^) for the *Macoma balthica* treatment.

	Coefficient	Intercept	*R* ^2^	*n*	*F*	*p*
ln Leaf area (mm^2^)	13.64	−39.26	.4	15	8.6	**.012**

*Note*: Bold indicates when significant (*p* < .05).

### 3.3 Community leaf tissue nitrogen relations to *M. balthica* condition index

**FIGURE 5:**
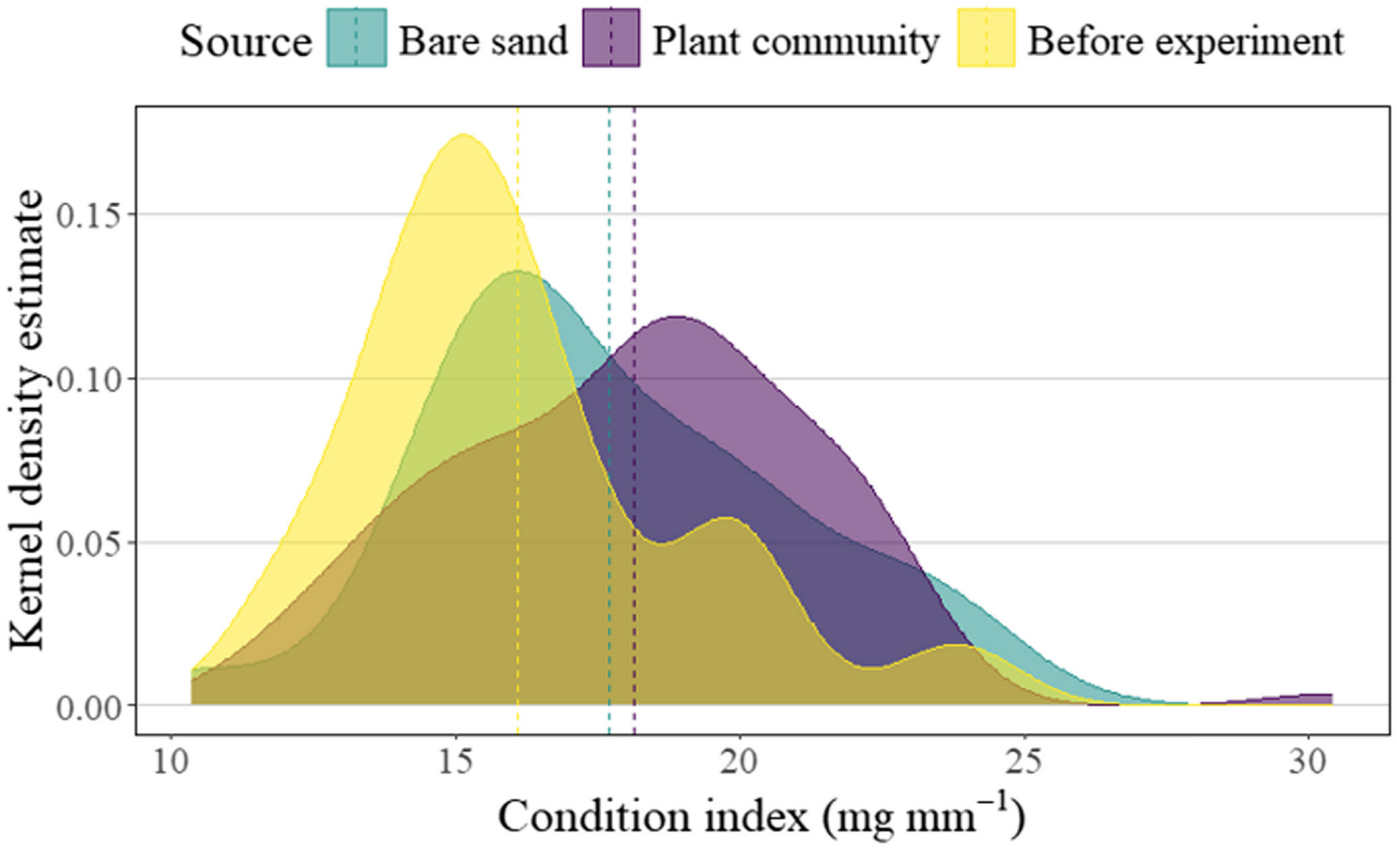
Density plot showing frequency distribution of *Macoma balthica* condition index (mg wet tissue/mm valve length) response to experimental transplantation in either a bare sand control treatment or an experimental plant community, in comparison to measured condition indices before the experiment commenced, in the Finnish archipelago of the northern Baltic Sea. Dashed lines show mean values.

The condition index (mg wet tissue/mm valve length) of *M. balthica* retrieved from vegetated plots significantly increased in rank, from measured condition indices prior to transplantation (Figure 5, *W* = 575, *p* < .01). Also, the modal condition index of *M. balthica* from the plant community treatment (30.4 mg mm^−1^) was considerably higher than the condition index for *M. balthica* retrieved from the bare sand treatment (24.04 mg mm^−1^) and from prior to transplantation (23.81 mg mm^−1^) (Figure 5). Given that condition indices from the bare sand treatment were intermediate to both the plant community treatment and prior to transplantation, they did not have a significant rank difference to condition indices from the plant community treatment (*W* = 179, *p* > .05), nor from prior to transplantation (*W* = 1113, *p* > .05).

**FIGURE 6:**
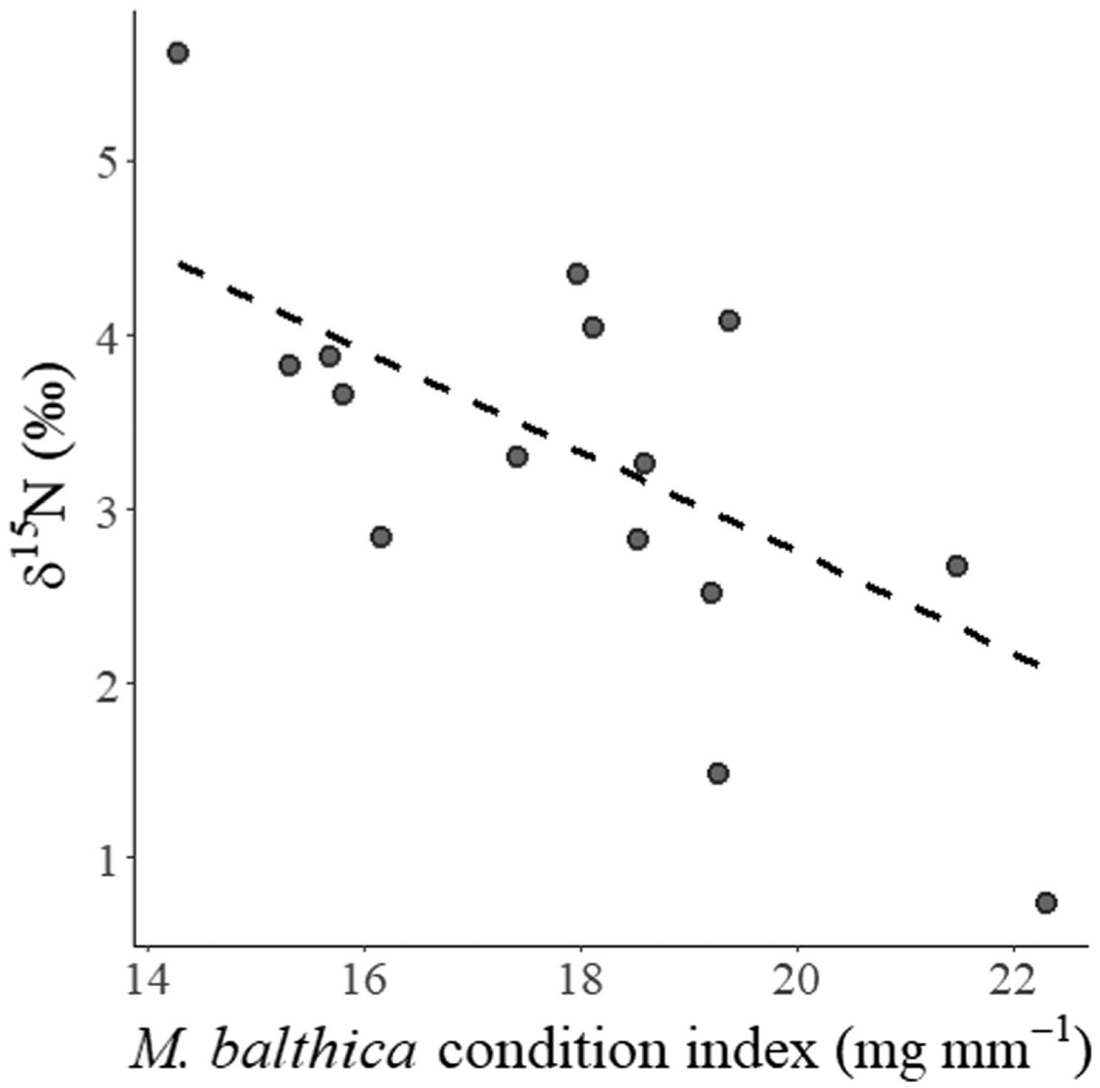
Community‐weighted mean leaf tissue nitrogen‐15 (δ^15^N, in relation to mean transplanted *Macoma balthica* condition index (soft tissue biomass [WW, mg]/valve length [mm]), for mixed species experimental plant communities in the Finnish archipelago, northern Baltic Sea. Dashed lines show the linear mixed model fit.

With increasing mean *M. balthica* condition index, community leaf tissue δ^15^N became significantly more depleted (Figure 6; Table 4). There was a 22% likelihood that δ^15^N measurements from the same experimental block would correlate (ICC = 0.22). There was no significant relationship between community leaf tissue N (% DW) and condition index of the retrieved *M. balthica* (Table 4).

**TABLE 4:** Linear mixed model fits for the relationships between plot mean *Macoma balthica* condition index (CI, soft tissue biomass [WW, mg]/valve length [mm]) and community‐weighted mean leaf tissue δ^15^N (‰) and N (% Dry Weight) (*n* = 15).

Regressor	Response	Intercept	Slope	*R* ^2^(*M*)	*R* ^2^(*C*)	ICC
*M. balthica* CI	δ^15^N	8.55 ± 1.71	−0.29 ± 0.09	.39	.61	0.22
	N (%)	1.23 ± 1.13	0.12 ± 0.06	.21	.42	0.21

*Note*: Pseudo‐*R* ^2^ values are as follows: The marginal *R* ^2^ (*R* ^2^(*M*)) is for CI effects, while the conditional *R* ^2^ (*R* ^2^(*C*)) is for the combined effects of CI and experimental block.

## 4 DISCUSSION

This study provides a unique insight into the instrumental role of *M. balthica* to plant community structure, and to the relationship between plant community traits and biomass production. An attribute of our experimental gradient approach was that experimental plant communities were likely more prone to exhibit trait responses to *M. balthica* because experimental CWMs varied along a gradient which increased the likelihood of observing changes in plant trait CWMs and plant trait‐productivity relationships. Our experiment shows that *M. balthica* additions did not significantly change total plant community biomass production. Instead, we observed relationships between a suite of traits and community productivity which suggested that clam additions changed the traits that characterised the most productive plant communities. This might be caused by intraspecific plasticity of trait manifestation as well as changes in relative species abundances (i.e., reduced *P. perfoliatus* biomass production) with *M. balthica* additions. Overall, this interaction suggests that *M. balthica* had changed the predominant growth strategy by the SAV (i.e., shift from height‐related to root trait‐related community biomass production).

### 4.1 Community biomass production sensitivity to experimental facilitation

Since N increases photosynthetic capacity (Evans, 1989; Evans & Clarke, 2019), we had hypothesised (H1) an increase in total community biomass production in response to *M. balthica* additions. Indeed, this was observed for seagrass mesocosms with hatchet shell (Lucinidae) additions (Cardini et al., 2022), and in salt marshes with gulf ribbed mussel (*Geukensia demissa*) density manipulations (Rossi et al., 2022). However, Hypothesis 1 was rejected, because there was not a significant increase in plant community biomass production. One potential reason for the low biomass response in our experiment, is that plants produce more biomass in response to nitrogen additions when there is more light available for photosynthesis (Harbur & Owen, 2004). Therefore, light availability could have limited a stronger biomass response in this experiment. After all, during the experimental period, the daily maximum photosynthetically active radiation ranged between only 168 and 555 μmol m^−2^ s^−1^ (Angove et al., 2020).

In addition to potential light limitation, another reason why the hypothesised increase in total community biomass production was not observed, is that there may not have been enough clams added to each plot to supersede the variability of natural abundances. However, our treatment increased the adult *M. balthica* population by 30%, which is expected to be functionally important (Norkko et al., 2013). Furthermore, in the northern Baltic Sea there is a risk that *M. balthica* additions can inhibit seagrass growth if they are too high for the environmental context (Meysick et al., 2020). Results from our study suggested that this risk was present in our plant communities, because the clam additions significantly inhibited growth of one plant species; *P. perfoliatus* (Figure 2). This could have been caused by reaching a species‐specific ammonium toxicity threshold (Touchette & Burkholder, 2000). Resultantly, a higher clam density addition may not have necessarily increased community biomass production and instead, it could have further accentuated species‐specific responses to higher sediment nutrient levels. As a consequence, infauna could affect the community structure of SAV by their selective effects on different species.

Our results showed that plants had likely facilitated *M. balthica* by increasing condition index from prior to the transplant (Figure 5). Therefore, it is likely that the SAV had nourished *M. balthica*, for example as a carbon source (Williams et al., 2009), and by entraining particles from the water column (Fonseca & Fisher, 1986). Accordingly, *M. balthica* could have also shifted from suspension‐feeding, towards more deposit‐feeding behaviour, since there is such plasticity in its feeding habits in response to hydrodynamic and biotic settings (Törnroos et al., 2015). This finding provides supporting evidence that habitat quality and food availability is a driver of *M. balthica* abundance, alongside other candidate factors (Bonsdorff et al., 1995; Seitz, 2011). Meanwhile, the bare sand treatment did not nourish *M. balthica* to the same extent, so that there was not a significant rank difference in condition index compared to before the transplant. As *M. balthica* condition index increased, plant community leaf tissue became significantly more depleted in δ^15^N (‰), as predicted by Hypothesis 4. This indicates that *M. balthica* excretions could have been a nitrogen source to plants because *M. balthica* metabolism would have selectively retained the heavy isotope ^15^N and excreted the lighter isotope ^14^N in a process of fractionation (Minagawa & Wada, 1984), making more ^15^N‐depleted nitrogen available for plants to absorb. Since plant δ^15^N (‰) responded to the physiological activity of transplanted *M. balthica*, the plants were potentially absorbing nitrogen as readily as it became available. Importantly, these results show that, while our experimental treatment was an addition to natural abundances of *M. balthica*, the plants were still sensitive enough to the transplanted mussels, that their leaf tissue δ^15^N nutrient concentrations responded to their physiological activity (i.e., Condition index).

### 4.2 Increased relationship between root traits and community biomass production

Communities with low mean SRLs; that is, thicker or denser roots, produced significantly more biomass. We thus rejected Hypothesis 2, which predicted a positive relationship between community SRL and plant community biomass production, because there was instead a significant negative relationship. A lower SRL facilitates roots with a greater force to penetrate the sediment, as well as a better capacity to transport solutes, a relatively slower growth and longer lifespan (Aerts, 1999; Eissenstat, 1992; Pérez‐Harguindeguy et al., 2013). Also, with increasing nutrient availability, particularly nitrogen (Ostonen et al., 2007), SRL can decrease because there is a reduced necessity for root exploration to find nutrient sources (Eissenstat, 1992; Ostonen et al., 2007). Therefore, while we expected that communities with higher SRL would produce more biomass in the *M. balthica* treatment, the reason for the observed reverse relationship is still well explained. Since the experiment was conducted for a large portion of the growing season, the short‐term benefits of finer roots could have been redundant considering that most plant growth could have proliferated in response to the *M. balthica* additions and not just fine roots.

Overall, our results showed that the plants which invested in longer and thicker/denser roots were significantly more productive in the *M. balthica* treatment, and this was not observed in the control treatment. Such responsiveness to the *M. balthica* treatment supports previous evidence that seagrass proliferates new shoots in sediments where there is a higher availability of nutrients (Furman et al., 2017), and that the belowground biomass of plants actively forages for nutrients in the sediment (Campbell et al., 1991; de Kroon & Mommer, 2006; Kembel et al., 2008). Indeed, individual plants in the studied archipelago can deplete nutrients in their surrounding sediments via a biomass‐driven demand (Angove et al., 2018). Therefore, it is understandable that there was a clear benefit to plants which invested in sediment nutrient acquisition in the *M. balthica* treatment, while it may not have benefited the control treatment, because a forage response may not have been stimulated. Nonetheless, the causal mechanism cannot be identified owing to the multiple effects that *M. balthica* have to their surrounding environment, including sediment porewater enrichment of nutrients, organic and inorganic carbon, and sediment oxygenation (Braeckman et al., 2010; Ebenhöh et al., 1995; Michaud et al., 2005; Norkko et al., 2013). Importantly, their effect to the surroundings of plants is relevant enough to change the plant traits that are most beneficial to plant productivity, thus they can change the drivers for SAV growth.

### 4.3 Decoupling of relationship between plant height and community productivity

Plant height is conventionally (Cadotte, 2017; Díaz et al., 2004) known to be fundamental for describing primary productivity in plants, and empirically known to be closely related to community productivity in temperate submerged aquatic plant meadows (Gustafsson & Norkko, 2019). It can have a disproportionally high effect on biomass production (Angove et al., 2020; Cardinale et al., 2002) because it can significantly increase the productivity of plants in addition to stimulating a competitive response (Angove et al., 2020). Therefore, despite the disproportional increase to biomass production that plant height can endow, the *M. balthica* treatment negated the relationship between community‐weighted height and plant biomass production, unlike in the control treatment. Therefore, we rejected Hypothesis 3, which predicted that both height and leaf area would be significantly related to biomass production in both the control and treatment. Our results exacerbate the scale of how much the plants could change their growth strategy in response to infaunal abundance. While one of the taller species in the assemblages (*P. perfoliatus*) had significantly inhibited growth (Figure 2), there was no overall significant difference in height CWMs between treatments (Table S2).

### 4.4 Outlook on facilitation under global change

The outcome of studies which test facilitation between SAV and infauna have notable importance in decision‐making practices for SAV conservation, because infauna could be highly important for future SAV restoration and resilience (Cardini et al., 2022; Clemente & Thomsen, 2023; Gagnon et al., 2020). This study is an example of how infauna have an integral role to SAV community productivity, via an alternative pathway to total community biomass production; plant‐trait productivity relationships. Therefore, if there is not a total community biomass response to infauna additions, there can still be fundamental changes to the SAV which change the plant traits that characterise high biomass production. The long‐term implications of this under the changing climate have yet to be discerned, but future research which explores the interplay between plant traits, productivity, infauna, and warming temperatures could help to explain the mechanisms behind future changes in facilitation with the changing climate. It is also important to consider the infauna response to future change. For instance, ongoing climate change is expected to reduce survival and reproduction of *M. balthica* by multiple mechanisms, including spawning mismatches, metabolic demands, and resource limitations (Beukema et al., 2009; Philippart et al., 2003). Their mismatch between resource availability and metabolic demand in response to warming temperatures has been observed via declines in their condition index (Beukema et al., 2009). In our study, SAV significantly increased *M. balthica* condition index, therefore SAV could have an important role to their survival and reproduction in a warming climate, by increasing their nutrient resource and mitigating declines in their condition.

## 5 CONCLUSION

Our study is unique for exploring the effects of infauna on SAV trait‐biomass production relationships. Its novel findings enrich our understanding of interactions between SAV and infauna, by showing that experimental *M. balthica* additions shifted the plant functional traits most closely related to SAV community biomass production. There became a stronger relationship between SAV biomass production and sediment‐nutrient acquisition traits and a decoupling between height and community biomass production. While our *M. balthica* treatment was an addition to natural abundances, the plants were still acutely sensitive to the physiological condition of transplanted individuals, because their new leaf tissue δ^15^N was significantly related to recaptured clam condition index. This highlights the critical role of *M. balthica* to the drivers of SAV growth, by changing the traits most closely related to biomass production, and it emphasises the importance of facilitation between SAV and infauna in SAV response to future change.

## AUTHOR CONTRIBUTIONS

**Charlotte Angove:** Conceptualization (equal). **Alf Norkko:** Conceptualization (equal). **Camilla Gustafsson:** Conceptualization (equal).

## FUNDING INFORMATION

This research was funded by the Walter and Andrée de Nottbeck Foundation (CA & CG), Research Council of Finland (AN, grant number 294853; CG, grant number 295443; CA, grant number 341984), Svenska Kulturfonden (CG), Kone Foundation (CA, grant number 202006108) and Societas pro Fauna et Flora Fennica (CA).

## CONFLICT OF INTEREST STATEMENT

All authors have no conflict of interest to declare.

## Supporting information


Data S1.

## Data Availability

All the necessary data has been uploaded as supplementary materials for the reviewers. Data will be made openly available in a public repository that issues datasets with DOIs.
