# Supplementary material for: Root puppet masters: Infauna shift trait‐productivity relationships in submerged aquatic vegetation communities
Source: Ecol Evol. 2024 Oct 25;14(10):e70305. doi: 10.1002/ece3.70305 (PMC11511661; doi:10.1002/ece3.70305)
Supplement: Supplementary file 1 — Data S1. [file ECE3-14-e70305-s001.docx]

**Supporting information**


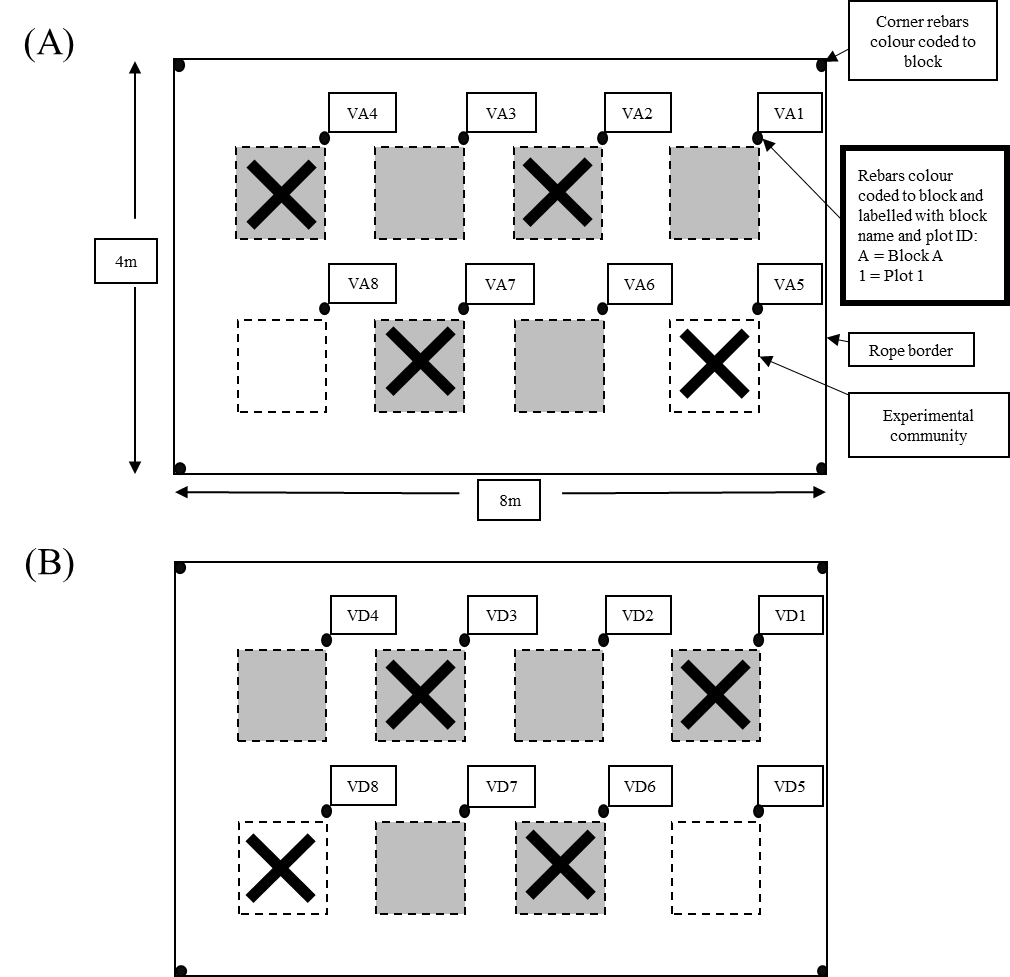


Figure S1. (A) Schematic design of one block of experimental plant communities. The grey boxes are artificial plant tricultures, while the white boxes are the bare sand treatment. Boxes marked with an ‘X’ were treated with the clam additions. (B) The design of a block paired with (A), with identical artificial plant assemblages but opposite clam additions.

| Blocks | Plot number | Triculture selection | | |
| --- | --- | --- | --- | --- |
| A & D | 1 | *Stuckenia pectinata* | *Potamogeton perfoliatus* | *Zannichellia major*  Table S1. Species combinations for triculture communities across experimental blocks. |
|  | 2 | *Stuckenia pectinata* | *Ruppia cirrhosa* | *Myriophyllum spicatum* |
|  | 3 | *Stuckenia pectinata* | *Zannichellia major* | *Ruppia cirrhosa* |
|  | 4 | *Zostera marina* | *Zannichellia major* | *Ruppia cirrhosa* |
|  | 5 | *-* | *-* | *-* |
|  | 6 | *Stuckenia pectinata* | *Potamogeton perfoliatus* | *Ruppia cirrhosa* |
|  | 7 | *Potamogeton perfoliatus* | *Zannichellia major* | *Ruppia cirrhosa* |
|  | 8 | *-* | *-* | *-* |
| B & E | 1 | *-* | *-* | *-* |
|  | 2 | *Zostera marina* | *Stuckenia pectinata* | *Potamogeton perfoliatus* |
|  | 3 | *Stuckenia pectinata* | *Potamogeton perfoliatus* | *Zannichellia major* |
|  | 4 | *Zostera marina* | *Stuckenia pectinata* | *Potamogeton perfoliatus* |
|  | 5 | *-* | *-* | *-* |
|  | 6 | *Zostera marina* | *Potamogeton perfoliatus* | *Zannichellia major* |
|  | 7 | *Potamogeton perfoliatus* | *Zannichellia major* | *Ruppia cirrhosa* |
|  | 8 | *Zostera marina* | *Stuckenia pectinata* | *Zannichellia major* |
| C & F | 1 | *Zostera marina* | *Potamogeton perfoliatus* | *Ruppia cirrhosa* |
|  | 2 | *Zostera marina* | *Zannichellia major* | *Myriophyllum spicatum* |
|  | 3 | *Stuckenia pectinata* | *Zannichellia major* | *Ruppia cirrhosa* |
|  | 4 | *-* | *-* | *-* |
|  | 5 | *Zostera marina* | *Stuckenia pectinata* | *Zannichellia major* |
|  | 6 | *Zostera marina* | *Stuckenia pectinata* | *Ruppia cirrhosa* |
|  | 7 | *Zostera marina* | *Stuckenia pectinata* | *Ruppia cirrhosa* |
|  | 8 | *-* | *-* | *-* |

| Table S2. T test statistics comparing mean plant trait community weighted means (CWM) between *Macoma balthica* treatments in this experiment, and control plots. Leaf area data was log-transformed and one extreme SRL value was removed to facilitate parametric comparison. | *t* | df | *p* |
| --- | --- | --- | --- |
| Height CWM (cm) | -1.447 | 25 | 0.16 |
| Leaf area CWM (mm^2^) | -0.905 | 26 | 0.37 |
| MMRL CWM (cm) | 0.165 | 25 | 0.87 |
| SRL CWM | -0.528 | 23 | 0.60 |
| N CWM (% DW) | 0.708 | 27 | 0.48 |
| δ¹⁵N CWM | -1.181 | 21 | 0.25 |
